# The Great Lakes Regional Stroke Network Experience

**Published:** 2006-09-15

**Authors:** Angela Bray Hedworth, Cassidy S Smith

**Affiliations:** Great Lakes Regional Stroke Network, Center for Stroke Research, University of Illinois at Chicago; Colorado Clinical Guidelines Collaborative, Lakewood, Colo. Ms. Smith was with the Great Lakes Regional Stroke Network, Center for Stroke Research, University of Illinois at Chicago, Chicago, Ill, at the time the article was written.

## Abstract

Stroke is a leading cause of disability and the third leading cause of death among adults in the United States and in the Great Lakes states of Illinois, Indiana, Michigan, Minnesota, Ohio, and Wisconsin. The Great Lakes Regional Stroke Network was created to enhance collaboration and coordination among the Great Lakes states to reduce the burden of stroke and stroke-related disparities associated with race, sex, and geography. Three priorities were identified for reducing the effects of stroke in the Great Lakes region: 1) build epidemiologic capacity to improve stroke prevention and control efforts, 2) facilitate systems-level changes and collaborative efforts to improve acute stroke care and rehabilitation, and 3) promote awareness of the warning signs of stroke and the need to call 911. The Great Lakes Regional Stroke Network has work groups in the areas of epidemiology and surveillance, health care quality improvement, and public education. These groups recommend initiatives to states for their efforts to reduce the effects of stroke within the Great Lakes region. Examples of recommended initiatives include identifying and prioritizing state research evaluation needs for stroke, conducting a stroke education media campaign, and developing a statewide emergency medical services protocol for stroke.

## Introduction

Stroke is the third leading cause of death in the United States and a leading cause of long-term disability (1). In the United States there are approximately 4.5 million stroke survivors (2), and according to the American Stroke Association (ASA), there are approximately 500,000 new strokes per year. On average, someone has a stroke every 45 seconds, and someone dies of a stroke about every 3 minutes. One third of strokes occur in people younger than 65 years (3). In an article in *Stroke*, Elkins and Johnston predict stroke deaths will increase 98% between 2002 and 2032 (4).

In 2004, the Centers for Disease Control and Prevention (CDC) funded a regional stroke network comprising the following Great Lakes states: Illinois, Indiana, Michigan, Minnesota, Ohio, and Wisconsin. One of the first tasks for the Great Lakes Regional Stroke Network (GLRSN) was to develop a document detailing stroke statistics for the Great Lakes region. In this area, stroke is a leading cause of long-term adult disability, is the third leading cause of death among adults, and accounted for 25,000 deaths, or 5.7% of all deaths, in 2002 (5).

According to estimates from the Behavioral Risk Factor Surveillance System (BRFSS) and other state-administered surveys, more than 880,000 people in the Great Lakes region live with the effects of stroke. In the Great Lakes region in 2002, the age-adjusted stroke mortality rate per 100,000 people ranged from 51.3 in Minnesota to 60.1 in Indiana; Illinois, Indiana, Michigan, Ohio, and Wisconsin have higher rates of stroke mortality than the U.S. age-adjusted rate of 56.4 per 100,000. These rates far exceed the *Healthy People 2010* objective of no more than 48 stroke deaths per 100,000 (6). Black men in all six GLRSN states had the highest age-adjusted stroke mortality rates overall with ranges from 74.6 in Wisconsin to 89.9 in Ohio per 100,000.

Modifiable risk factors for stroke are prevalent in the Great Lakes region. In 2003, Indiana, Michigan, and Ohio had higher percentages of adults with diabetes, high cholesterol levels, and high blood pressure who also smoked, were obese, and had an unhealthy diet compared to the U.S. median. Illinois had a higher percentage of adults with diabetes and obesity and who had an unhealthy diet (7). (Additional statistics about the region are available from the GLRSN Web site at http://glrsn.uic.edu.)

In 2003, CDC published *A Public Health Action Plan to Prevent Heart Disease and Stroke* (8). One of the components of this comprehensive action plan is to encourage the public health community to engage in regional and global partnerships to increase stroke prevention resources and capitalize on shared experiences. The ASA's Task Force on the Development of Stroke Systems also described a need for effective interaction and collaboration among health care professionals, services, and agencies that treat stroke (9). In 2002, a need for greater coordination and support mechanisms among health care professionals was also mentioned by a task force sponsored by the National Institute of Neurological Disorders and Stroke (10).

CDC recognized the importance of collaboration by state heart disease and stroke prevention programs and funded three stroke networks. The GLRSN is the most recently funded network (2004). Other stroke networks funded by CDC include the Tri-State Stroke Network (established in the late 1990s to coordinate stroke efforts in North Carolina, Georgia, and South Carolina) and the Delta States Stroke Consortium (funded in 2002 to coordinate efforts in Alabama, Arkansas, Louisiana, Mississippi, and Tennessee). The GLRSN benefited from the experiences of previous networks and adapted their models to the needs of the Great Lakes region.

## GLRSN Priorities and Work Assignments

The role of the GLRSN is to increase stroke awareness, prevention, and control activities across the Great Lakes region (Illinois, Indiana, Michigan, Minnesota, Ohio, and Wisconsin). These states have partnered for more than 25 years on other cardiovascular disease initiatives. Formation of the GLRSN presented a challenge for states to consider initiatives that could affect change at a systems level. Internal structure of the GLRSN includes a structure work group (initial stage), a steering committee, a state advisory board, work groups that address the three priority areas of epidemiology and surveillance, improved quality of care, and public education, and state task force committees.

### Structure work group

A structure work group was identified to create the infrastructure needed for the GLRSN. This group created a policy and procedures manual to be updated as needed with items such as job descriptions for work group leads, information about how decisions are made, and conflict resolution steps. After an initial year of meetings, this group decided to streamline GLRSN activities and combine its meetings with those of the state advisory board.

### Steering committee

The steering committee is composed of heart disease and stroke prevention program staff, stroke task force liaisons, representatives from partner organizations (e.g., ASA, the National Stroke Association [NSA]), a CDC project officer, and priority work group representatives. This group meets via conference call two to three times each year. The group's focus is professional development, and calls feature a professional presentation from a stroke-related agency, such as the Brain Attack Coalition, the Commission on Accreditation of Rehabilitation Facilities, or the Association of Black Cardiologists. A strategic planning session of the state advisory board found there is a need to enhance this committee with additional professional organizations.

### State advisory board

The GLRSN state advisory board includes state heart disease and stroke prevention program managers and the steering committee. The state advisory board had its first face-to-face meeting in February 2005 to develop a work plan to address coordination and collaboration efforts to reduce the effects of stroke in Great Lakes states. The state advisory board meets once a year and is the GLRSN decision-making body responsible for strategic planning and setting priorities. At a recent strategic planning session, the board recommended enhancing the steering committee by seeking involvement with additional professional organizations.

### Priority work groups

The GLRSN identified the following three priority areas for its efforts to reduce the effects of stroke in the Great Lakes region: epidemiology and surveillance, improved quality of care, and public education. For each priority area there is a work group comprising individuals from each state. State heart disease and stroke prevention program managers identify a representative for each work group, and groups communicate by conference call.

**Epidemiology and surveillance**

The goal of the epidemiology and surveillance work group is to build epidemiologic capacity to improve stroke prevention and control efforts. The group determined that the following projects would help them meet this goal: 1) identify and prioritize stroke research and evaluation funding, 2) create a single document detailing the effects of stroke within the Great Lakes Region, 3) develop stroke fact sheets for each state, 4) collaborate with the GLRSN work group focused on improved quality of care to ensure uniform data collection across the region, and 5) organize a data exchange to be held in Chicago in December 2006 to discuss innovative stroke research projects in each of the Great Lakes states.

**Improved quality of care**

The goal of the work group focused on improved quality of care is to facilitate systems-level changes and collaborative efforts to improve acute stroke care and rehabilitation. The following activities were identified to meet this goal: 1) conduct an assessment of emergency medical services (EMS) in collaboration with the state EMS agency to determine capacity to handle stroke emergencies, 2) develop or improve statewide EMS stroke protocols to include use of a stroke scale or clinical assessment tool (e.g., Cincinnati Stroke Scale, Los Angeles Prehospital Stroke Screen, or other emergency assessment) to identify neurological deficits, 3) promote appropriate stroke emergency training for dispatchers and first responders, 4) conduct a stroke training module at state EMS conferences, 5) collaborate with state quality improvement organizations on training initiatives about stroke prevention and care, 6) promote communication among rehabilitation specialists and managed care organizations to coordinate stroke patient care effectively, 7) invite rehabilitation specialists and managed care organizations to participate in state stroke task force committees, and 8) share successful stroke protocols (hospital, physician, and EMS) with the GLRSN.

The group working on improved quality of care reviewed findings from the Paul Coverdell National Acute Stroke Registry prototypes in Michigan, Ohio (11), and Illinois (12) and from the Center for Medicare and Medicaid Services findings in the *Sixth Scope of Work* stroke measures (13). These reviews revealed that improvements are needed in the following areas: 1) deep vein thrombosis prophylaxis, 2) lipid profiles, 3) coordination of atrial fibrillation treatment with anticoagulation therapy, 4) dysphagia screening, 5) smoking cessation counseling, and 6) physician, EMS personnel, and public education about the urgency of stroke and the short time after a stroke that tissue plasminogen activator (tPA) treatment can be given to some patients to reverse stroke effects.

The quality improvement group shared with GLRSN states a list of quality improvement tools, resources about evidence-based clinical guidelines, and stroke registry quality improvement templates from the Illinois Care and Prevention Treatment Utilization Registry (CAPTURE) program. The group assembled a panel of EMS professionals to discuss EMS stroke initiatives with GLRSN partners. Future work group projects include sharing stroke rehabilitation resources among GLRSN states and cosponsoring workshops about improving stroke quality of care with the National Stroke Association.

**Public education**

The goal of the public education work group is to promote awareness of the warning signs of stroke and the need to call 911. The group identified the following activities to meet this goal: 1) implement strategies to reach high-risk populations with messages about stroke symptoms, 2) explore partnership opportunities with major professional sports teams to create stroke public education events, 3) conduct stroke awareness activities annually in May and include events such as a proclamation by the governor to declare May as stroke awareness month, 4) conduct a stroke education media campaign using public service announcements or paid advertisements, and 5) partner with state agencies, such as offices of bioterrorism and EMS, to discuss expanded access to 911 and enhanced 911 services.

These activities require partnerships among state health departments, the American Heart Association (AHA), ASA, NSA, and other public health stakeholders. The public education work group has completed an inventory of stroke public education events in all GLRSN states, prepared a resource list of available stroke public education and media tools, and created a document, *Working With Professional Sports Teams: How to Do a Stroke Public Education Event*, that was distributed through the GLRSN Web site to members and other interested groups and through the listserv for CDC cardiovascular state programs. The goal of this document is to assist heart disease and stroke prevention programs and partners to organize stroke education programs with the asset of visibility that comes from working with professional sports teams. 

### State stroke task force committees

Each state developed a stroke task force committee if one did not already exist, and states had different experiences because of varying legislative requirements, organizational structures, and financial and staff resources. States were given financial resources to begin and sustain a task force for three years. Michigan has a stroke task force in place voluntarily, and Ohio, Indiana, and Illinois have legislatively mandated stroke task forces. Wisconsin and Minnesota developed stroke task forces after receiving funding from the GLRSN.

The purpose of each task force is to implement recommendations developed by work groups in the priority areas of epidemiology and surveillance, quality of care improvement, and public education. Stroke task force committees assist in providing direction for state systems-level change and have been integral to the development of the GLRSN.

In states that receive CDC heart disease and stroke prevention program funding, the stroke task force works closely with the state heart disease and stroke prevention coalition. Stroke task force activities include 1) development of treatment guidelines for stroke in Indiana, 2) in Wisconsin, the creation of two continuing medical education programs about treatment of stroke and stroke center certification by the Joint Commission on Accreditation of Health Care Organizations (JCAHO), and 3) in Michigan, development of a fact sheet, *Understanding Your Health Care Benefits for Stroke Rehabilitation*. Each state stroke task force committee is instrumental in implementing recommendations from GLRSN work groups.

## Successes

The GLRSN has carried out several successful projects since its inception, and these include an inventory that is the first step in completing a regional stroke plan for Great Lakes states. This comprehensive inventory of each state will enable GLRSN states to better understand their capabilities in the following areas: 1) EMS and stroke care, 2) state legislation related to stroke, 3) stroke risk factors, 4) acute stroke treatment, 5) stroke rehabilitation, and 6) state stroke task force committees.

Other GLRSN achievements include fostering partnerships among its states and organizations, such as the AHA, ASA, NSA, and other national organizations, and presenting posters at the International Stroke Conference and the Stroke Belt Consortium. The GLRSN has excelled at sharing stroke-related experiences and resources among its states through its Web site, listserv, monthly e-bulletin, and conference calls.

## Barriers to Implementation

Because the structure of each state health department is unique and both financial and personnel resources vary widely, the GLRSN has limited ability to implement some activities. Two GLRSN states do not receive CDC funding for state heart disease and stroke prevention programs.

The format and amount of stroke-specific data vary among states. After several attempts, the GLRSN was unable to find and share comparable state-specific quality improvement data for stroke because this information is almost nonexistent in the Great Lakes region. Several hospitals in the region have limited staff and funds for implementing stroke quality-of-care improvement tools. As a result, data that do exist are not complete representations of the state. Stroke mortality data by race were limited because hospitals are not required to report data on race and ethnicity, and population estimates on race and ethnicity are unreliable. Not all states conducted the heart disease and stroke module in the BRFSS survey, and this difference resulted in variations of available data. A variety of stroke education materials are used by GLRSN states, and there is no consistent message or evaluation for these materials.

## Going Forward

The GLRSN identified the following elements as necessary to continue developing and enhancing a regional approach to reduce the effects of stroke: 1) a public education message must be developed so that a consistent message about stroke symptoms and response is presented across the region; 2) a systematic, regional approach to data collection and analysis is needed to assess the scope of the regional effects of stroke; 3) stroke quality-of-care improvement initiatives that can benefit the region as a whole should be explored and implemented; and 4) financial sustainability for the GLRSN must be achieved to enable the network to continue its mission of collaboration and coordination among Great Lakes states to reduce the burden of stroke and stroke-related disparities associated with race, sex, and geography.

## Conclusion

The GLRSN is a regional partnership of state heart disease and stroke prevention programs, community partners, national organizations, and state stroke task force groups. The goals of the GLRSN are to increase stroke awareness and prevention activities across the Great Lakes region and to enhance collaboration and coordination among states to reduce the effects of stroke. The GLRSN operates through work groups that focus on three priority areas: epidemiology and surveillance, quality-of-care improvement, and public education. Recommendations from these work groups are presented to each state stroke task force for review and consideration so that the task force can select and implement recommendations as resources allow.

## References

[1] (2001). American Heart Association. Noteworthy numbers from the 2002 statistical update.

[2] Casper ML, Barnett E, Williams GI, Halverson JA, Braham VE, Greenlund KJ (2003). Atlas of stroke mortality: racial, ethnic and geographic disparities in the United States.

[3] (2005). National Stroke Association Rapid Response Task Force. National Stroke Association stroke rapid response.

[4] Elkins JS, Johnston SC (2003). Thirty-year projections for deaths from ischemic stroke in the United States. Stroke.

[5] Kochanek KD, Murphy SL, Anderson RN, Scott C (2004). Deaths: final data for 2002. National vital statistics reports.

[6] (2000). U.S. Department of Health and Human Services. Healthy people 2010. With understanding and improving health.

[7] (2006). Great Lakes Regional Stroke Network. The burden of stroke in the Great Lakes states.

[8] (2003). U.S. Department of Health and Human Services. A public health action plan to prevent heart disease and stroke: executive summary and overview.

[9] Schwamm LH, Pancioli A, Acker JE, Goldstein LB, Zorowitz RD, Shephard TJ (2005). Recommendations for the establishment of stroke systems of care: recommendations from the American Stroke Association's Task Force on the Development of Stroke Systems. Stroke.

[10] (2002). National Institute of Neurological Disorders and Stroke Symposium. Task force report. Improving the chain of recovery for acute stroke in your community (publication no. 03-5348).

[11] (2005). The Paul Coverdell Prototype Registries Writing Group. Acute stroke care in the U.S.: results from four pilot prototypes of the Paul Coverdell National Acute Stroke Registry. Stroke.

[12] (2006). Illinois information from Dilip Pandey, oral communication.

[13] Jencks SF, Huff ED, Cuerdon T (2003). Change in the quality of care delivered to Medicare beneficiaries, 1998-1999 to 2000-2001. JAMA.

